# Interleukin-25 and eosinophils progenitor cell mobilization in allergic asthma

**DOI:** 10.1186/s13601-018-0190-2

**Published:** 2018-02-13

**Authors:** Wei Tang, Steven G. Smith, Wei Du, Akash Gugilla, Juan Du, John Paul Oliveria, Karen Howie, Brittany M. Salter, Gail M. Gauvreau, Paul M. O’Byrne, Roma Sehmi

**Affiliations:** 1 Division of Respirology, Department of Medicine, McMaster University, Hamilton, ON L6M 1A6 Canada; 2 Department of Respirology and Critical Medicine, Ruijin Hospital, Shanghai Jiaotong University School of Medicine, Shanghai, China

**Keywords:** Asthma, Allergen challenge, Eosinophils progenitor, IL-25, IL-25 receptors, BrdU, CD34

## Abstract

**Background:**

Eosinophil-lineage committed progenitor cells (EoP) migrate from the bone marrow and differentiate locally to provide an ongoing source of mature eosinophils in asthmatic inflammatory responses in the airways. Sputum levels of EoP are increased in asthmatics compared to normal controls suggesting an exaggerated eosinophilopoietic environment in the airways. Understanding what factors promote EoP traffic to the airways is important to understand the diathesis of asthma pathology. Interleukin (IL)-25, is an epithelial-derived cytokine that promotes type 2 inflammatory responses. We have previously shown that levels of IL-25 and expression of the IL-25 receptor (IL-17RA and IL-17RB) on mature eosinophils are greater in allergic asthmatics compared to atopic non-asthmatics and non-atopic normal controls. In addition, these levels were increased significantly increased following allergen inhalation challenge and physiologically relevant levels of IL-25 stimulated eosinophil degranulation, intracellular IL-5 and IL-13 expression and primed migration to eotaxin. The current study, examined the role of IL-25 on allergen-induced trafficking of EoP in atopic asthmatics.

**Methods:**

Asthmatics (n = 14) who developed allergen-induced early and late responses were enrolled in the study. Blood was collected at pre- and 24 h post-challenge. At each time point, surface expression of IL-17RA and IL-17RB on EoP was evaluated by flow cytometry. Migration assays examined the effect of IL-25 on EoP chemotactic responses, in vitro. In addition, IL-25 knockout ovalbumin (OVA) sensitized and challenged mice were studied to evaluate in vivo mobilization effects of IL-25 on newly formed EoP and mature eosinophils.

**Results:**

There was a significant increase in numbers of blood EoP expressing IL-17RB, 24 h post-allergen inhalation challenge in allergic asthmatics. Pre-exposure to IL-25 primed the migrational responsiveness of EoP to stromal cell-derived factor 1α. In OVA-sensitized mice, knocking out IL-25 significantly alleviated OVA-induced eosinophil infiltration in the airway and newly formed eosinophils were reduced in the lung.

**Conclusions:**

The findings of this study indicate a potential role for IL-25 in allergen-induced trafficking of EoP to the airways and local differentiation promoting tissue eosiniophilia in asthmatic responses.

**Electronic supplementary material:**

The online version of this article (10.1186/s13601-018-0190-2) contains supplementary material, which is available to authorized users.

## Background

Asthma is a chronic disease of the airways characterized by reversible airflow obstruction, airway inflammation and airway hypperesponsiveness. Tissue eosinophilia and type 2 cytokine producing cells including T-helper (Th) 2 cells and group 2 innate lymphoid cells, are the predominant components of the airway inflammatory cell infiltrate in subjects with allergic asthma [1].

Interleukin-25 (IL-25; IL-17E) is a pro-inflammatory cytokine that belongs to the IL-17 cytokine family and, unlike other members of the IL-17 family, plays a pivotal role in the maintenance of type 2 immune responses [2]. IL-25 has been shown to directly activate eosinophils, by up-regulation of the adhesion molecule ICAM-1, stimulate the release of pro-inflammatory chemokines such as monocyte chemoattractant protein-1, IL-8, macrophage inflammatory protein-1 and IL-6, as well as delay apoptosis [3, 4]. The IL-25 receptor consists of two subunits, IL-17RA (the signaling sub-unit) and IL-17RB (the specific cytokine binding subunit) that form the functional heterodimeric receptor, IL-17RA/RB [5]. In a previous baseline cross-sectional study, we have shown significantly increased expression of IL-17RA and IL-17RB on mature eosinophils and plasma levels of IL-25 in asymptomatic mild allergic asthmatics compared with atopic non-asthmatics and non-atopic normal subjects [6]. In addition, we reported significant increases in plasma levels of IL-25 and intracellular IL-25 eosinophil levels, as well as IL-17RA/RB and IL-17RB receptor expression on mature eosinophils, 24 h following allergen-inhalation challenge in allergic asthmatics [7]. Furthermore in vitro experiments showed that at physiologically relevant concentrations, IL-25 stimulated eosinophil degranulation and primed the migrational responses of mature eosinophils.

A considerable body of evidence supports the view that in allergic asthma, eosinophil-lineage committed progenitor cells (EoP) traffic from the bone marrow to the lungs via the peripheral circulation and that the local tissue-driven differentiation of these cells may contribute to the development and maintenance of tissue eosinophilia [8]. The effect of IL-25 on the traffic of bone marrow-derived hemopoietic progenitor cells (HPC) and more specifically eosinophil-lineage commmited progenitor cells (EoP) to the lungs in allergic asthmatic responses has not been reported to date.

In this study, we examined IL-25 and IL-25R expression on EoP in asthmatic subjects following allergen-inhalation challenge. In addition, we employed an OVA-sensitized mouse model to investigate whether traffic of mature eosinophils and newly formed eosinophils to the site of inflammation was influenced by IL-25.

## Methods

### Study design

Fourteen subjects with mild allergic asthma, aged between 19 and 52 years, were enrolled in the study. All volunteers were atopic with one or more positive skin prick tests; a forced expired volume in 1 s (FEV_1_) greater or equal to 70% of predicted; and dual airway responses to inhaled allergen as determined by a fall in FEV_1_ ≥ 15% within the first 2 h, followed by second fall in FEV_1_ between 3 and 7 h after allergen inhalation challenge (Table 1). All subjects were steroid naïve and only intermittently used β_2_-agonists. Subjects attended the laboratory for three consecutive visits. On visit 1 (day 1), a medical history and physical examination were performed and subjects underwent a skin prick test, spirometry, methacholine inhalation challenge. On visit 2 (day 2), subjects underwent allergen inhalation challenge and spirometry was measured hourly up to 7 h post-challenge. At Visit 3 (day 3), spirometry and methacholine challenge was performed 24 h after inhalation challenge. Flow cytometric assessments were performed on blood samples collected before and 24 h post-allergen challenge. All subjects gave written informed consent, and the study was approved by the Hamilton Health Science Research Ethics Board (HIREB # 12-583).

**Table 1:** Baseline subject characteristics

Sex	Age	Ag inhaled	%FEV1 (%predicted)	PBaseline PC_20_ (mg/ml)
M	19	Cat	81	0.31
M	25	HDM	114	10.31
F	19	Cat	117	20.80
F	49	HDM	97	1.07
M	44	Grass	73	16.00
F	41	Ragweed	94	0.83
F	47	Cat	90	0.60
F	20	Tree	92	3.19
M	52	HDM	100	6.99
M	27	Ragweed	97	2.71
F	21	Horse	98	16.00
F	19	Ragweed	80	5.82
M	49	HDM	90	16.00
F	24	HDM	116	0.38
F	25	HDM	108	15.48

Subject characteristics: all subjects were skin prick test positive; had a forced expired volume in 1 s (FEV1) ≥ 70% predicted; FEV_1_—forced expiratory volume in 1 s; all patients PC_20_—provocative concentration of methacholine causing a 20% drop in FEV_1_; *HDM* house dust mite; *Ag* allergen

### Allergen inhalation challenge

Allergen inhalation was performed as previously described [9]. The allergen producing the largest diameter skin wheel was diluted in saline for inhalation. The concentration of allergen required to achieve a 20% decrease in FEV_1_ (the allergen PC_20_) was predicted using the methacholine PC_20_ and the titration of allergen determined from the skin prick test. The early asthmatic response (EAR) was recorded as the greatest fall in FEV_1_ between 0 and 2 h after allergen inhalation, whereas the greatest drop in FEV_1_ between 3 and 7 h was recorded as the late asthmatic response (LAR) as previously described [9].

### Cell preparation and Immunofluorescence staining

For identifying CD34^+^ hemopoietic progenitor cells, 20 mL heparinized venous blood was diluted with McCoys 5A (Invitrogen, USA), then layered on Lymphoprep (Axis-Shield, USA) and centrifuged (2200 rpm, 20 min). Mononuclear cells were removed and washed with McCoy’s 5A (centrifugation at 1500 rpm for 10 min at 4 °C) and re-suspended in FACS buffer, then immunostained with CD34-Alexa Fluor 700, CD45-Pacific Blue, CD125-APC, IL17RA-FITC and IL17RB-PE or corresponding isotype controls (BD Bioscience and R&D systems). Cells were incubated for 30 min at 4 °C then washed with FACS buffer and fixed in 1% PFA prior to flow cytometric acquisition.

### Flow cytometry acquisition

Data were acquired using a 15-color LSR II flow cytometer equipped with 3 lasers (Becton–Dickinson Instrument Systems) with FACSDiva software program (Becton–Dickinson Biosciences). Following acquisition of 300,000 events, data analyses were performed using FlowJo software version 9.3.2. (Tree Star Inc.) to enumerate IL-25 receptor components expression; for gating strategy details see Additional file 1: Fig. S1 for hemopoietic progenitor cells (HPC; CD34^high^CD45^dull^) and eosinophil-lineage committed progenitor cells (EoP; CD34^high^CD45^dull^CD125^high^) as previously described in detail [10].

### Progenitor cell migration—trans well migration assay

The migrational response of progenitors was assessed in transwell chambers (24-well cell clusters, 6.5 mm Transwell^®^ with 5 µm pore polycarbonate membrane insert filters; Corning Costar, NY, USA) as previously described [10]. Briefly, peripheral blood mononuclear cells isolated, as described above, were depleted by adherence to plastic (2 h, 5% CO2 and 37 °C) and CD34^+^ cells enriched by positive selection using MACS immunomagnetic beads (Miltenyi Biotec, CA, USA). Cell purity of CD34^+^ cells was > 95%, viability > 90%. The chemoattractant, SDF-1 (CXCL12; 10 ng/mL; R&D Systems) or diluent (IMDM plus 10% FBS) was loaded into the lower well and CD34^+^ cells (5 × 10^4^) were added to the upper transwell inserts. Cells in the lower well (representing migrated responding cells) were immunostained as HPC (CD34^+^CD45^+^) or EoP (CD45^+^CD34^+^CD125^+^) and enumerated by flow cytometry, as described above. Migrated cells were expressed as a percentage of the total cells added to the top transwell. Cells were pre-incubated with IL-25 (R&D Systems) for 18 h (37 °C, 5% CO_2_) prior to the migration assay.

### OVA sensitized mice

Wild type C57BL/6 mice were purchased from Shanghai SLAC laboratory Co., Ltd. (Shanghai, China). IL-25 knockout mice on the same background were purchased from Qinghua University animal center (Beijin, China). Mice were aged between 6 and 8 weeks-old, weighing between 20 and 22 g, and were housed at 18–25 °C, humidity 50–60%, 0.03% CO_2_, 12/12 h light/dark cycle and food/water were available ad libitum and refreshed every 3 days. For both wild type and IL-25KO mice, the sensitized animals (OVA sensitized and OVA challenged; n = 6) received an intraperitoneal injection of 100 µg ovalbumin (OVA; Sigma Aldrich; Merck KGaA) and 2 mg alum (Sigma Aldrich; Merck KGaA) in PBS on days 0, 7 and 14. On days 25, 26 and 27, the mice were challenge with aerosolized 1% OVA in PBS for 30 min. For both wild type and IL-25KO mice, Sham control mice (Sham sensitized and Sham challenged, n = 6) received PBS intraperitoneally with alum on days 0, 7 and 14, and were challenged with aerosolized PBS on days 25, 26 and 27. BrdU (1 mg) was given by intraperitoneal injection twice per day, on days 25 and 27, 30 min prior to challenge with OVA as previously described [11, 12].

#### Bronchoalveolar lavage fluid (BALF), blood and bone marrow sampling

BALF collection was performed 24 h after the final OVA or PBS challenge. Lungs were lavaged with 1 mL PBS through the trachea and BALF was collected. Blood (0.5–0.8 mL) was drawn prior to BALF collection into heparin from each mouse. Bone marrow was harvested from the femur and tibia into heparin as previously described [12]. Smears of blood and bone marrow samples were made to perform eosinophil counts following standard staining with hematoxolin and eosin.

#### Measurement of eosinophil number in BALF

Cells were seeded in PBS medium (Beyotime Institute of Biotechnology, Haimen, China) at 1 × 10^5^ cells/mL and stained with Fast Wright and Giemsa Stain kit (Nanjing Jiancheng Technology Co., Ltd., Nanjing, China), according to manufacturer’s protocol. Eosinophils were counted with a light microscope and expressed as percent eosinophils.

### Inflammatory index measurement

The lung inflammation index score after OVA or diluent challenge was measured as previously described [13]. The index is: 0 equals no inflammatory infiltration; 1 equals a minimal inflammatory cell infiltration; 2 equals a layer of inflammatory cells annular infiltration; 3 equals 2–4 layers of inflammatory cell ring infiltration; 4 equals more than 4 layers of inflammatory cells annular infiltration.

### Immunohistochemistry (IHC) semi-quantitative analysis

The expression levels of BrdU in lung tissues were quantified by Image-Pro Plus 6.0, expressed as mean optical density (OD), which equals intensity of optical density divided by area of lung tissues observed under microscope (400×). Newly produced eosinophils were identified as BrdU positive and eosin positive cells.

### Statistical analysis

Statistical analysis was performed using GraphPad Prism5 software (GraphPad Software Inc.). This study is powered on sputum eosinophil progenitor cells in mild asthmatics, and changes 24 h post-allergen were the primary outcome. Assuming within subject variability from previously published data in mild asthmatics [9], the sample size required to detect the “minimal important differences” between baseline and 24 h post-allergen measurements was calculated. Based on repeated measures ANOVA analyses, using the sample size module of NCSS statistical package with β = 0.20 (power = 80%) and α = 0.05 (likelihood of type 1 error = 5%) and a minimum important difference = 0.72, SD = 0.59, the sample size is calculated to be n = 14. Normally distributed data are expressed as mean ± SEM. The methacholine PC_20_ is expressed as geometric mean and geometric standard error of the mean (GSEM). For HPC and EoP IL-25 receptor expression and migration experiments, a repeated measures ANOVA was used. Post-hoc comparisons were performed using the Tukey’s multiple comparison tests. p < 0.05 was considered significant for all analyses.

## Results

**Fig. 1:**
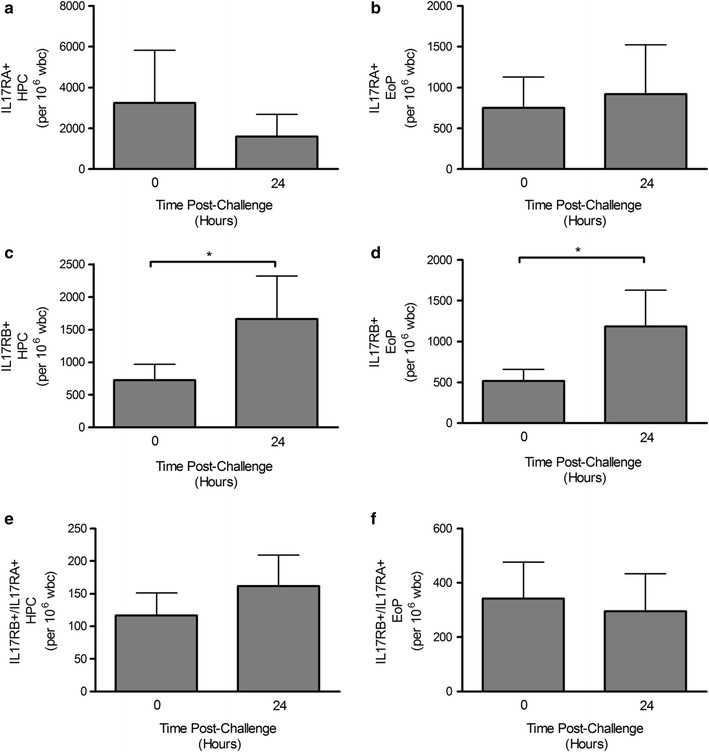
Allergen-induced changes in IL-25 receptor expression on blood HPC and EoP. Expression of IL-17RA^+^ (**a**, **b**), IL-17RB^+^ (**c**, **d**) and IL-17RA/RB^+^ (**e**, **f**) in mild allergic asthmatics following allergen-inhalation challenge. There was a significant increase in the number of HPC and EoP expressing IL-17RB^+^ 24 h post-allergen inhalation challenge. Data are mean ± SEM (n = 14)

Following allergen inhalation challenge, all asthmatic subjects developed an early and late bronchoconstrictor responses; the maximal early fall in FEV_1_ (within 0–2 h post-allergen) was 33.67 + 8.22% and the maximal late fall in FEV_1_ (3–7 h post-allergen inhalation) was 22.47 + 8.43% (Table 2). This was associated with a significant increase airway eosinophilia (Table 2) and total number of number of blood HPC and EoP (HPC: 1663 ± 657 vs. 723 ± 244 per 10^6^ WBC, p < 0.01; EoP: 1188 ± 442 vs. 519 ± 140 per 10^6^ WBC, p < 0.01) 24 h post-allergen compared to pre-allergen levels. Further phenotypic analyses of HPC and EoP demonstrated significant increases in expression of IL-25 specific binding sub-unit (IL-17RB), 24 h post-allergen compared to pre-allergen baseline levels (Fig. 1). In contrast, these changes were not observed for the signaling sub-unit IL-17RA or combined receptor complex IL-17RA/RB on either HPC or EoP (Fig. 1).

**Table 2:** Subject lung function and sputum

	Allergen challenge
EAR (% change in FEV_1_)	− 33.67 ± 2.12^#^
LAR (% change in FEV_1_)	− 24.47 ± 1.77^#^
Methacoline PC_20_ (mg/mL)	
Pre-Ag	7.77 ± 1.88
24 h post-Ag	2.20 ± 0.51*
Total sputum cells (×10^6^ cells/mL)	
Pre-Ag	3.62 ± 0.64
24 h Post-Ag	6.59 ± 1.27*
Sputum eosinophils (%)	
Pre-Ag	4.68 ± 1.63
24 h Post-Ag	12.26 ± 2.68*^,#^
Blood eosinophils (per 10^9^ WBC)	
Pre-Ag	38 ± 6
24 h Post-Ag	64 ± 9*

Data are presented as geometric mean ± SEM. There was a significant difference the EAR and LAR % change in FEV_1_, methacoline PC_20_, total sputum cells, and sputum and blood eosinophils post-allergen

*FEV*_*1*_ forced expiratory volume in 1 s; *PC20* provocative concentration of methacholine causing a 20% drop in FEV_1_, *Ag* allergen, *EAR* early asthmatic response, *LAR* late asthmatic response, *WBC* white blood cells, *HPC* hemopoietic progenitor cells, *EoP* eosinophil progenitors

*p < 0.05 comparison to baseline and ^#^p < 0.05 comparison to diluent

**Fig. 2:**
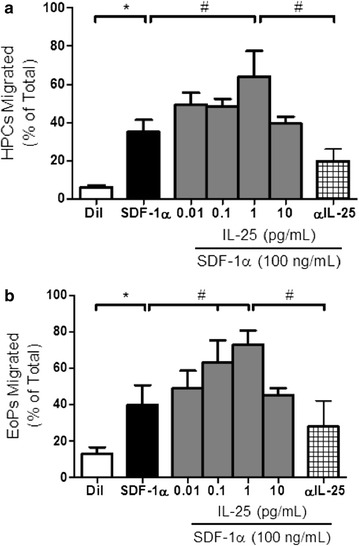
IL-25 priming of (**a**) HPC and **b** EoP migration, in vitro. Pre-incubation overnight with IL-25 primed the migrational responsiveness of both HPC and EoP stimulated by SDF-1α. SDF-1α alone induced migration. Data are presented as mean ± SEM (n = 6) (*p < 0.05 comparison to diluent; ^#^p < 0.05 comparison to SDF-1α alone)

In pilot studies, we found that IL-25 over a wide concentration range (0.1 – 1000 pg/ml) has no effect on the migrational responses of EoP compared to diluent control, in vitro (data not shown). However, pre-incubation with IL-25 (optimal concentration 1 pg/ml), compared to diluent, significantly enhanced the subsequent migrational response of both HPC and EoP to a sub-optimal concentration of SDF-1α (10 ng/ml) (HPC: 64 ± 13 vs. 35 ± 6 and EoP: 73 ± 8 vs. 39 ± 10 respectively, p < 0.0001) (Fig. 2). Pre-treatment with an IL-25 neutralizing antibody (previously optimized at 2.4 ng/ml) significantly inhibited the priming effect of IL-25 on SDF-1α stimulated migration of HPC and EoP (Fig. 2).

**Fig. 3:**
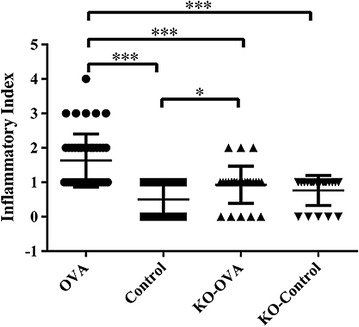
Inflammatory index of lung tissues from wild type and IL-25 KO mouse models that were sensitized and challenged with OVA or PBS (control) (*p < 0.05 and ***p < 0.001, n = 5)

**Fig. 4:**
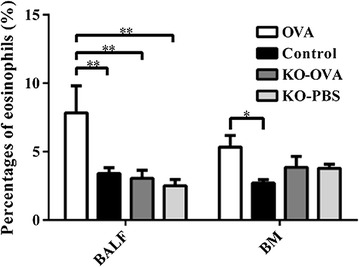
Eosinophil percentage of BALF, blood and bone marrow from wild type and IL-25 KO mouse models that were sensitized and challenged with OVA or PBS (control) (*p < 0.05 and **p < 0.01, n = 5)

In IL-25 knock out (KO) mice that were OVA-sensitized, a partial but significant attenuation of airway inflammation following allergen -challenge was observed (Additional file 2: Fig. S2). The inflammatory index in the control mice compared to wild type OVA challenged and IL-25 KO OVA challenged mice were 0.50 ± 0.51 vs. 1.63 ± 0.77 vs 0.93 ± 0.54 respectively (p < 0.001 for all comparisons to control mice) (Fig. 3). Furthermore, OVA challenge significantly increased the percentages of eosinophils in wild type compared to control mice in BALF (7.83 ± 4.84 vs. 3.40 ± 0.43%) and bone marrow samples (5.33 ± 0.85 vs. 2.7 ± 0.25%) (Fig. 4). Compared to the wild type mice, eosinophils levels were significantly attenuated in the OVA challenged IL-25 KO mice in BALF (7.83 ± 4.84, vs. 3.06 ± 1.78%, p < 0.01) (Fig. 4).

**Fig. 5:**
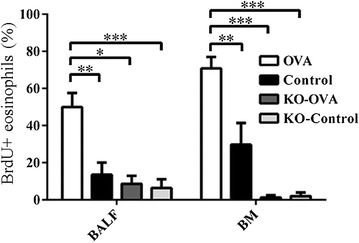
Percentage of BrdU positive eosinophils of BALF, blood and bone marrow from wild type and IL-25 KO mouse models that were sensitized and challenged with OVA or PBS (control) (*p < 0.05, **p < 0.01 and ***p < 0.001, n = 5)

**Fig. 6:**
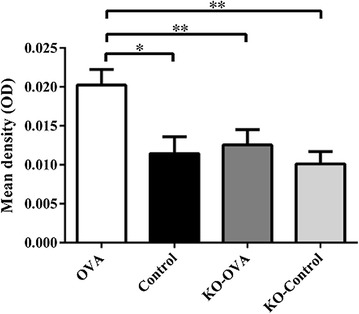
Expression of BrdU measured by immunohistochemistry wild type and IL-25 KO mouse models that were sensitized and challenged with OVA or PBS (control) (*p < 0.05, **p < 0.01 and ***p < 0.001, n = 5)

The proportion of BrdU + eosinophils expressed as a percentage of the total eosinophils in BAL and bone marrow was significantly reduced in OVA challenged IL-25 KO mice compared to wild type mice (BAL: 12.5 ± 6.51 vs. 51.7 ± 7.56%, p < 0.01; bone marrow: 2.4 ± 1.28 vs. 69.1 ± 6.11%, p < 0.001) (Fig. 5). In the lungs, BrdU expression after OVA challenge was significantly decreased in IL-25 KO mice compared with wild type mice (mean density, 0.013 ± 0.010OD vs. 0.020 ± 0.013OD; p < 0.01), (Fig. 6).

## Discussion

This study has demonstrated that following allergen-inhalation challenge in allergic asthmatic subjects, IL-17RB is up-regulated on the surface of HPC, as well as EoP in the blood. In addition, IL-25 primes migrational responses of blood-derived HPC and EoP to the progenitor chemoattractant, SDF-1α. These findings suggest that in response to inhaled allergen, upregulation of IL-25 receptor binding sub-unit expression on EoP in peripheral blood may promote increased occupancy of IL-25 on its specific receptor and stimulate priming of migrational responsiveness, thereby facilitating the homing of eosinophil precursors to the airways. Furthermore, in OVA-sensitized mice, knocking out IL-25 significantly alleviated lung inflammation, airway eosinophil infiltration and lung homing of newly produced eosinophils.

The biological effects of IL-25 are mediated by IL-25 receptor, which is composed of sub-units IL-17RA and IL-17RB. IL-25 is the high affinity ligand for IL-17RB, while IL-17RA shares the common ligand for IL-17A [5, 14]. Polymorphisms in the IL-17RB gene in humans have been linked with asthma susceptibility [15]. In a previous study, we have shown that IL-25 receptor expression on eosinophils is markedly higher in allergic asthmatics compared with atopic non-asthmatic and normal subjects [6]. In addition, we showed that the level of plasma IL-25 significantly increases following allergen inhalation challenge in allergic asthmatics [7]. In humans, IL-25 is produced by structural cells, such as epithelial and endothelial cells, and inflammatory cells, such as eosinophils, basophils and mast cells. IL-25 has been shown to link innate and adaptive immunity by enhancing type-2 cytokine production, including IL-5 and IL-13 [2].

The current study suggests that IL-25 plays a role in the recruitment of immature eosinophils to the airways in asthma. Our previous research has shown that EoP traffic from the systemic circulation into inflamed tissue sites, the migration orchestrated by locally produced chemokines, such as SDF-1α [16]. In line with these findings, our current data demonstrate that, although IL-25 did not directly stimulate migrational responses of EoP, pre-exposure to IL-25 enhanced the subsequent migrational response to SDF-1α. As such, it can be postulated that IL-25 may contribute to eosinophilic inflammation observed in the lung following allergen exposure through the priming of migrational responses of immature and mature eosinophils.

We have previously shown a significant increase in HPC and EoP in the sputum 24 h post-allergen challenge, which was associated with a significant increase in the expression of receptors for epithelial derived cytokines including TSLP (TSLPR and CD127) and IL-33 (ST2) on HPC and EoP [10]. Furthermore, pre-exposure to TSLP and IL-33 primed the migration of progenitor HPC and this effect was inhibited by blocking antibodies to TSLPR and ST2, respectively, suggesting that lung-homing of HPC maybe orchestrated by epithelial-derived cytokines, including TSLP and IL-33 [10]. Our current data support the view that the epithelial-derived cytokine IL-25 can prime the migrational response of these cells and promote lung-homing. In contrast to findings with TSLP and IL-33, we show here for the first time that IL-25 can prime the migrational responses of HPC *and* EoP while the latter cytokines only had effects on HPC [10]. A limitation of the study was that we performed these receptor up-regulation analyses in the blood as opposed to sputum samples as have been described in the above mentioned study. However, our findings in blood-derived eosinophil progenitor cell populations were similar to changes in sputum. Furthermore, the priming experiments with IL-25 were in agreement with the priming experiments performed with TSLP and IL-33 in blood derived cells suggesting similarity in the underlying mechanism.

The pro-inflammatory effects of IL-25 has been well demonstrated in animal models. Exogenous administration of IL-25, or transgenic expression induces type 2 asthma-like inflammation in the airways in mice [17, 18]. Conversely, anti-IL-25 antibody reduces airway inflammation in animal models of allergic asthma [19, 20]. In addition, IL-25-deficient mice have significant suppression of the number of eosinophils and the levels of pro-inflammatory mediators in bronchoalveolar lavage fluids (BALF) [21]. In this current study, OVA challenge of sensitized IL-25-deficient mice not only decreased mobilization of mature eosinophils, but also newly formed eosinophils. In IL-25 KO mice, the attenuation of newly produced eosinophils (BrdU + eosinophils) was observed the airways (BALF) and bone marrow samples suggesting that IL-25 may be involved in the formation of newly produced eosinophils in the bone marrow, as well as in the airways. We acknowledge that by labeling with BrdU, this study only enumerated newly formed eosinophils and not EoP per se. As such, it is unclear as to whether these newly formed eosinophils matured within the bone marrow and migrated to the airways, or if EoP migrated to the airways and differentiated locally within the tissue to mature eosinophils. However, we have previously shown that EoP traffic to the site of inflammation and have the potential of forming eosinophils in situ [22, 23] thus supporting the view that the BrDU + eosinophils arose as a result of local differentiative processes.

## Conclusions

In summary, IL-25 high affinity receptor part (IL-17RB) expression on EoP is increased in the peripheral blood of subjects with asthma after allergen challenge. IL-25 also enhanced the migrational response of eosinophil progenitors. IL-25 knockout mice showed decreased eosinophilic inflammation in the bone marrow and airways. Finally, in IL-25 KO mice there was decreased mobilization of newly produced eosinophils after OVA challenge. These results suggest that increases in IL-25 and expression of its receptor on EoP are important in the trafficking of these cells from the bone marrow to the airways during allergen-induced airway responses in asthma and that IL-25 may be a useful drug target to attenuate allergen-induced airway responses.

## Additional files

**Additional file 1: Figure S1.** Sequential Multi-gating strategy: to identify hemopoietic progenitor cells (HPC: CD45^+^CD34^+^) and Eosinophil-lineage committed progenitors (EoP; CD45^+^CD34^+^CD125^+^) in blood using FlowJo software on a LSRII. FlowJo plots of specific antibody and isotype control antibody for IL-17RA and IL-17RB on (A) HPC and (B) EoP with a 98% confidence limit. Absolute cell numbers were calculated by multiplying the % positive quadrant static (upper right quadrant) with the absolute progenitor cell count and expressed per million white blood cells.

**Additional file 2: Figure S2.** Histological analysis of lungs. Hematoxylin-eosin staining of the C57BL/6 in wild type and IL-25 KO mouse models that were sensitized and challenged with OVA or PBS (control).

## Abbreviations

Ag: allergen
PB: peripheral blood
FEV_1_: forced expired volume in 1 s
PC_20_: concentration required to achieve a 20% decrease in FEV_1_
EAR: early asthmatic response
LAR: late asthmatic response
ELISA: enzyme-linked immunosorbent assay
HPC: hemopoietic progenitor cell
EoP: eosinophil-lineage committed progenitor cell
SDF-1α: stromal cell derived factor 1α
IL-25(IL-17E): interluekin-25
OVA: ovalbumin
BM: bone marrow
IHC: immunohistochemistry
BrdU: bromodeoxyuridine
KO: knock out
HE: hematoxylin–eosin
