# Supplementary figures and images for: Interleukin-25 and eosinophils progenitor cell mobilization in allergic asthma
Source: Clin Transl Allergy. 2018 Feb 13;8:5. doi: 10.1186/s13601-018-0190-2 (PMC5809891; doi:10.1186/s13601-018-0190-2)

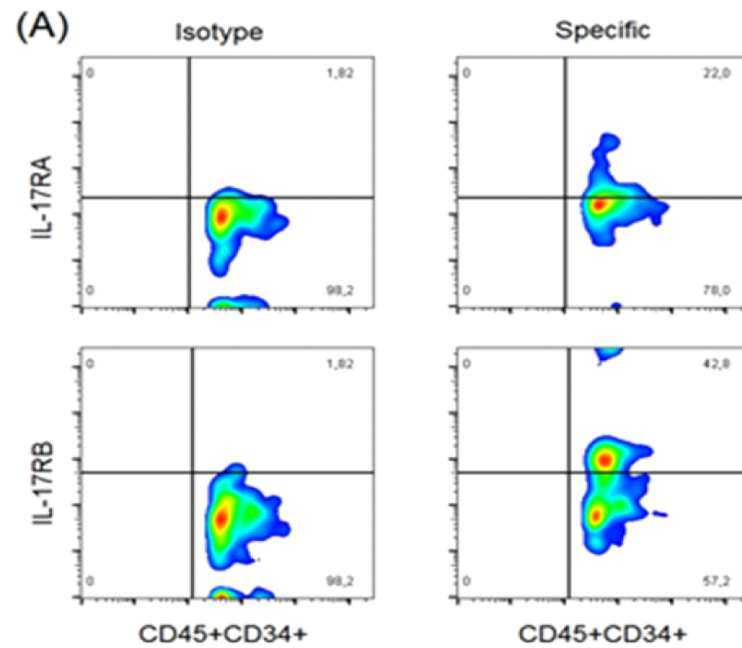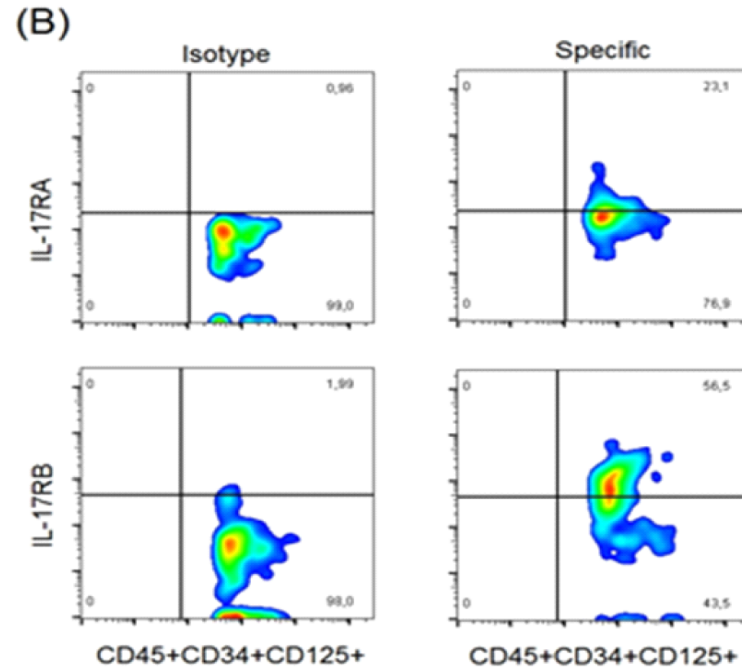

Supplement: Supplementary file 1 — Additional file 1: Figure S1. Sequential Multi-gating strategy: to identify hemopoietic progenitor cells (HPC: CD45+CD34+) and Eosinophil-lineage committed progenitors (EoP; CD45+CD34+CD125+) in blood using FlowJo software on a LSRII. FlowJo plots of specific antibody and isotype control antibody for IL-17RA and IL-17RB on (A) HPC and (B) EoP with a 98% confidence limit. Absolute cell numbers were calculated by multiplying the % positive quadrant static (upper right quadrant) with the absolute progenitor cell count and expressed per million white blood cells. [file 13601_2018_190_MOESM1_ESM.pdf]

**OVA**

**Control**

**Wild  
type**

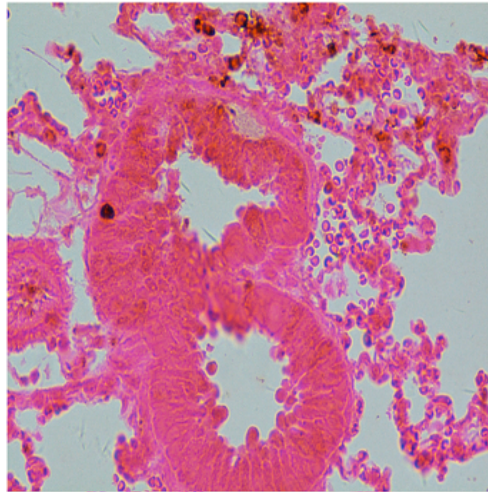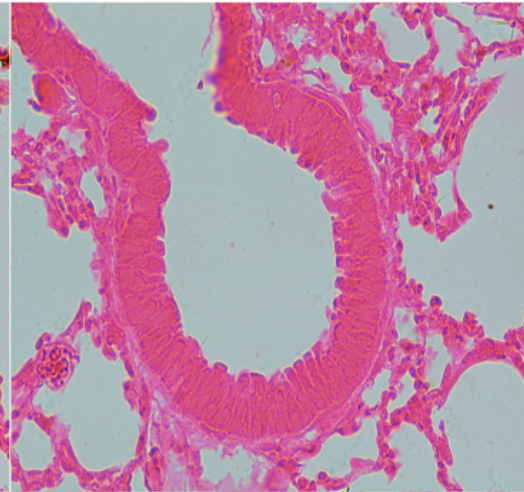

**Knock  
out**

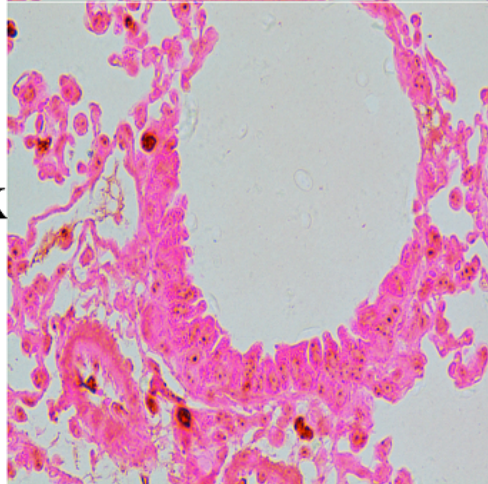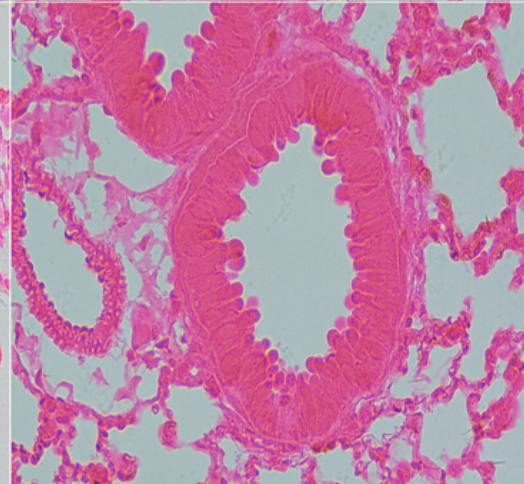

Supplement: Supplementary file 2 — Additional file 2: Figure S2. Histological analysis of lungs. Hematoxylin-eosin staining of the C57BL/6 in wild type and IL-25 KO mouse models that were sensitized and challenged with OVA or PBS (control). [file 13601_2018_190_MOESM2_ESM.pdf]
